# Stunting Is Characterized by Chronic Inflammation in Zimbabwean Infants

**DOI:** 10.1371/journal.pone.0086928

**Published:** 2014-02-18

**Authors:** Andrew J. Prendergast, Sandra Rukobo, Bernard Chasekwa, Kuda Mutasa, Robert Ntozini, Mduduzi N. N. Mbuya, Andrew Jones, Lawrence H. Moulton, Rebecca J. Stoltzfus, Jean H. Humphrey

**Affiliations:** 1 Centre for Paediatrics, Blizard Institute, Queen Mary University of London, London, United Kingdom; 2 Zvitambo Institute for Maternal Child Health Research, Harare, Zimbabwe; 3 Department of International Health, Johns Hopkins Bloomberg School of Public Health, Baltimore, Maryland, United States of America; 4 University of Michigan, School of Public Health, Ann Arbor, Michigan, United States of America; 5 Division of Nutritional Sciences, Cornell University, Ithaca, New York, United States of America; University of Washington, United States of America

## Abstract

**Background:**

Stunting affects one-third of children in developing countries, but the causes remain unclear. We hypothesized that enteropathy leads to low-grade inflammation, which suppresses the growth hormone-IGF axis and mediates stunting.

**Methods:**

We conducted a case-control study of 202 HIV-unexposed Zimbabwean infants who were stunted (height-for-age Z-score (HAZ) <−2; cases) or non-stunted (HAZ >−0.5; controls) at 18 months. We measured biomarkers of intestinal damage (I-FABP), inflammation (CRP, AGP, IL-6) and growth hormone-IGF axis (IGF-1, IGFBP3) in infant plasma at 6 weeks and 3, 6, 12 and 18 months, and in paired maternal-infant plasma at birth. Adjusted mean differences between biomarkers were estimated using regression models. Multivariate odds ratios of stunting were estimated by logistic regression.

**Results:**

At birth, cases were shorter (median (IQR) HAZ −1.00 (−1.53, −0.08) vs 0.03 (−0.57, 0.62,); P<0.001) than controls and their mothers had lower levels of IGF-1 (adjusted mean difference (95%CI) −21.4 (−39.8, −3.1) ng/mL). From 6 weeks to 12 months of age, levels of CRP and AGP were consistently higher and IGF-1 and IGFBP3 lower in cases versus controls; IGF-1 correlated inversely with inflammatory markers at all time-points. I-FABP increased between 3–12 months, indicating extensive intestinal damage during infancy, which was similar in cases and controls. In multivariate analysis, higher log_10_ levels of CRP (aOR 3.06 (95%CI 1.34, 6.99); P = 0.008) and AGP (aOR 7.87 (95%CI 0.74, 83.74); P = 0.087) during infancy were associated with stunting. There were no associations between levels of I-FABP, IL-6, sCD14 or EndoCAb and stunting.

**Conclusions:**

Stunting began *in utero* and was associated with low maternal IGF-1 levels at birth. Inflammatory markers were higher in cases than controls from 6 weeks of age and were associated with lower levels of IGF-1 throughout infancy. Higher levels of CRP and AGP during infancy were associated with stunting. These findings suggest that an extensive enteropathy occurs during infancy and that low-grade chronic inflammation may impair infant growth.

## Introduction

Stunting is an intractable public health problem affecting around one-third of children in developing countries [1]. Stunting underlies 14–17% of child deaths globally [1] and causes long-term cognitive defects, fewer years and poorer performance at school, lower adult economic productivity and an increased risk of stunting into subsequent generations [2]. Poor linear growth begins *in utero*, continues during the first 2 years of life and is largely irreversible thereafter [3]. Despite its high prevalence, the reasons for stunting among children living in impoverished conditions remain uncertain. Although inadequate diet contributes to poor growth, the best nutritional interventions have only a modest impact on stunting [4]. Diarrhea has been implicated in the causal pathway to stunting but, possibly because children frequently show catch-up growth between diarrheal episodes [5], the association has been surprisingly weak in many studies [6], [7], [8]. The role of the gut in mediating stunting has been relatively overlooked [9] until recently, when attention has refocused on the possible contribution of enteropathy to poor growth in early life [10].

It was recognized almost 50 years ago that people living in impoverished conditions almost universally had an abnormality of the small intestine, characterized by villous blunting, inflammatory infiltrate and increased intestinal permeability [11], [12]. This apparently asymptomatic condition was termed tropical enteropathy, although it became clear that environmental conditions, rather than geography *per se*, were the critical determinant [13]. It is hypothesized that frequent exposure to feco-oral bacteria in conditions of poor sanitation and hygiene drives a T-cell mediated process that has been renamed environmental enteropathy (EE) [14]. In the Gambia, researchers went on to show that the extent of EE, as measured by dual-sugar absorption testing, correlated inversely with linear growth during infancy [15]. It is hypothesized that EE enables intestinal bacteria and microbial-associated macromolecules to translocate across an impaired intestinal barrier to the systemic circulation, where they provoke immune activation, which impairs linear growth [10].

Stunting may therefore be driven by intestinal damage and chronic inflammation in addition to dietary inadequacy. Furthermore, since stunting begins *in utero*, the maternal inflammatory environment may have an important influence on fetal growth. However, few longitudinal studies have evaluated the mechanisms underlying poor growth among infants in developing countries. We hypothesized that an important cause of child stunting is exposure to chronic, low-grade inflammation during fetal and postnatal life, which suppresses production of IGF-1 [16], [17], perturbing the growth hormone axis early in life. We designed a case-control study to test this hypothesis, utilizing data and samples from a Zimbabwean birth cohort.

## Materials and Methods

### Ethics Statement

The original ZVITAMBO trial and this case-control study were approved by the Medical Research Council of Zimbabwe, Johns Hopkins Bloomberg School of Public Health Committee on Human Research, and Montreal General Hospital Ethics Committee.

### ZVITAMBO trial

We designed a case-control study using archived plasma samples and data from HIV-negative mother-infant pairs enrolled in the ZVITAMBO vitamin A supplementation trial, conducted between 1997–2001. The trial tested the effects of a single high-dose vitamin A supplement given to mothers and/or infants during the immediate postpartum period on several infant health outcomes, as previously reported [18]. Briefly, 14110 mother-infant pairs were enrolled within 96 h of delivery from clinics in Harare, Zimbabwe. Mother-infant pairs were eligible if neither had an acutely life-threatening condition, the infant was a singleton with birth weight ≥1500 g, and the mother planned to stay in Harare after delivery. Written informed consent was obtained. Socioeconomic and demographic information was collected by maternal interview. Follow-up was conducted at 6 w, 3 mo, then 3-monthly to 12–24 mo. At each visit, infant morbidity data were collected by maternal recall. Free medical care was provided throughout the trial.

### Infant feeding counseling and household sanitation practices

HIV-negative mothers were encouraged to exclusively breastfeed for six months. Data on feeding practices obtained at 6 w and 3 mo were used to categorize exclusive, predominant or mixed breastfeeding, as previously described [19]. At the time of the study, most households in Harare had sewage connection and tap water, but some people lived in informal settlements without these amenities and nearly all Zimbabweans regularly spend substantial periods of time in their rural homestead, especially during planting and harvesting seasons.

### Maternal and infant anthropometry

Maternal height and mid-upper arm circumference (MUAC) were measured within 96 h of delivery; maternal weight was measured at 6 w postpartum. Infant weight and height were measured using an electronic scale (Seca Model 727, Hanover, MD, USA), and length board (ShorrBoard, Olney, MD, USA), respectively, at each visit. Anthropometry was undertaken using methods described by Gibson [20]. Weight-for-age (WAZ), height-for-age (HAZ), and weight-for-height (WHZ) Z-scores were calculated based on the WHO 2010 reference standard using WHO Anthro version 3.0.1 (http://www.who.int/childgrowth/en). Among HIV-negative mothers, there was no effect of vitamin A supplementation on infant linear growth (Adetayo Omoni, unpublished data).

### Biological specimen collection

Blood was collected from all enrolled mothers and infants at baseline and from a representative subsample (52% of total) of mother-infant pairs at all follow-up visits. Samples were centrifuged and plasma removed within 2 h of blood collection; aliquots were stored in −80C freezers with automatic generator backup. Mothers underwent HIV testing at baseline using two parallel ELISA assays. Women who tested HIV-negative at baseline were re-tested at every subsequent visit to detect HIV seroconversion.

### Selection of cases and controls

Cases (stunted) and controls (non-stunted) for this study were selected based on HIV-exposure status and height at 18 mo of age. Eligible infants were born to mothers who remained HIV-negative throughout follow-up, and for whom anthropometric data and archived plasma of sufficient volume (≥0.2 mL) at ≥4 time-points between 6 w and 18 mo of age were available. Cases had height-for-age Z-score <−2.0, and controls had height-for-age Z-score >−0.5 at 18 mo.

**Figure 1:**
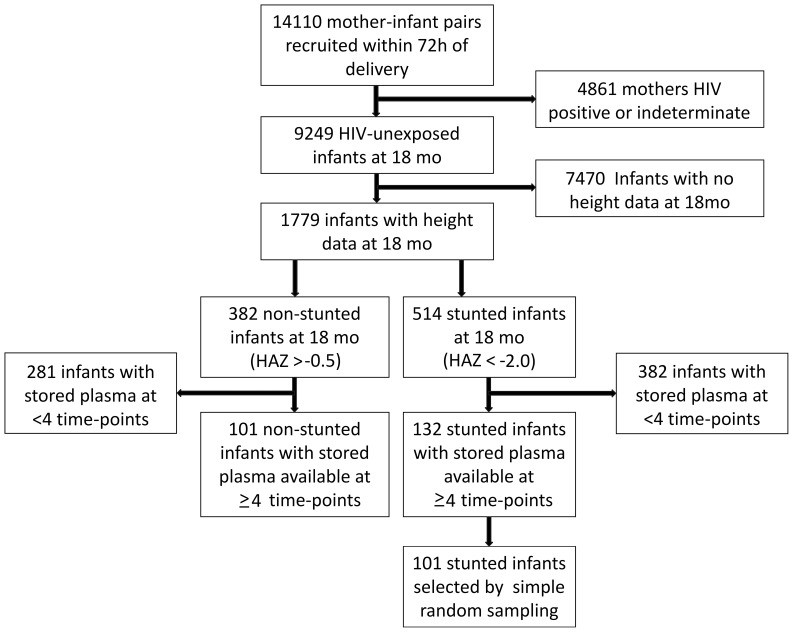
Selection of cases and controls. 14110 women were recruited within 96–2000. Eligible infants for this current study were born to women who were HIV-negative at baseline and remained uninfected through 18 months, who had anthropometry data available at 18 mo and stored plasma samples of ≥0.2 mL volume from at least 4 study time-points between 6 weeks and 18 months of age. Stunted infants were selected based on height-for-age Z-score <−2.0 at 18 mo; non-stunted infants were selected based on height-for-age Z-score >−0.5 at 18 mo.

Of 14110 enrolled infants, 9249 remained HIV-unexposed by 18 mo of age. From these, 132 stunted and 101 non-stunted infants fulfilled our selection criteria at 18 mo (Fig. 1). To maximize our sample size, we used all 101 controls, and randomly selected 101 cases from the stunted group. Plasma samples were available for all children at 18 mo, and for 75%, 85%, 91% and 97% at 6 w and 3, 6, 12 mo, respectively. We subsequently retrieved birth samples for a subgroup of infants (32%) with available stored plasma.

### Measurement of plasma biomarkers

We used commercially available ELISA kits to assess small intestinal damage (intestinal fatty acid binding protein [I-FABP; Hycult Biotechnology, Uden, The Netherlands]); microbial translocation (soluble CD14 [sCD14; R&D Systems] and IgG EndoCAb [Hycult Biotechnology]); immune activation (interleukin-6 [IL-6; R&D Systems], C-reactive protein [CRP; R&D Systems, Inc., Minneapolis, MN, USA] and alpha-1 acid glycoprotein [AGP; R&D Systems]); and activity of the growth hormone axis (insulin-like growth factor 1 (IGF-1; R&D Systems) and insulin-like growth factor binding protein 3 (IGFBP3; R&D Systems]).

### Statistical analysis

Baseline characteristics were compared between cases and controls using Chi-squared tests for categorical variables, Wilcoxon rank sum tests for continuous non-normal variables, or two-sample t-tests for continuous normal variables. Because of baseline differences in important covariates for stunting between cases and controls, adjusted differences in plasma biomarker concentrations were estimated at each time-point. For normally distributed biomarkers, unadjusted differences between means were compared using the two-sample t-test; adjusted differences between means were calculated using an ordinary least-squares regression model. For non-normally distributed biomarkers, unadjusted differences between medians were compared using the cendif command in STATA [21]; adjusted differences between medians were calculated using a median regression model. The covariates used to calculate adjusted differences in infant biomarkers were gender, birth weight, maternal MUAC and maternal education. The covariates used to calculate adjusted differences in maternal biomarkers were maternal height, weight, MUAC and education. Univariate and multivariate odds ratios for stunting were estimated for each predictor variable by logistic regression using covariates described in the text, selected on the basis of biological plausibility. Spearman correlations were undertaken to explore associations. Statistical analyses were performed using STATA version 10 (Stata-Corp, College Station, TX, USA) and Prism version 5 (GraphPad Software Inc., La Jolla, CA, USA).

### Study data

Data from this study are not available in a public repository but may be made available upon request.

## Results

### Baseline characteristics of infants and mothers

Baseline characteristics of infants and mothers are shown in Table 1. Infants who were stunted at 18 mo of age (cases) were more likely to be male than infants who were not stunted at 18 mo of age (controls) and were already lighter and shorter at birth. Despite promotion of exclusive breastfeeding, the majority of infants were mixed breastfed within the first 3 mo of life, which was more common among cases than controls (67% vs 51%, respectively; P = 0.022). Mothers of cases were lighter and shorter than mothers of controls, though only 3% and 7%, respectively, were underweight (BMI<18.5 kg/m^2^); 28% of mothers of cases and 39% of mothers of controls were overweight or obese (BMI>25 kg/m^2^). Mothers of infants who became stunted had fewer years of education than mothers of non-stunted infants.

**Table 1:** Baseline characteristics of infants and mothers.

Infant characteristics	Cases (stunted) N = 101	Controls (non-stunted) N = 101	P value
Male sex, % (n)	61 (62)	39 (39)	0.001
Gestational age, weeks; mean (SD)	39.0 (1.3)	39.5 (1.3)	0.018
Birth weight, kg; mean (SD)	2.85 (0.45)	3.17 (0.44)	<0.001
Low birth weight, % (n)*	23 (23)	7 (7)	0.002
WAZ at birth, median (IQR)	−1.00 (−1.73, 0.41)	−0.22 (−0.84, 0.35)	<0.001
WAZ at 18 months, median (IQR)	−1.53 (−2.09, −0.98)	0.36 (−0.01, 1.00)	<0.001
Birth length, cm; mean (SD)	47.9 (2.3)	49.4 (2.5)	<0.001
HAZ at birth, median (IQR)	−1.00 (−1.53, −0.08)	0.03 (−0.57, 0.62)	<0.001
HAZ at 18 months, median (IQR)	−2.66 (−3.00, −2.30)	0.27 (0.06, 0.66)	<0.001
WHZ at birth, mean (SD)	−0.65 (1.68)	−0.47 (1.48)	0.444
WHZ at 18 months, mean (SD)	−0.34 (1.20)	0.37 (1.23)	<0.001
Head circumference at birth, cm; mean (SD)	33.9 (1.5)	34.4 (1.3)	0.021
Head circumference at 18 months, cm; mean (SD)	47.3 (5.5)	47.9 (1.6)	0.298
Normal vaginal delivery, % (n)	90 (91)	94 (95)	0.297
Apgar score at 5 minutes, median (IQR)	10 (9, 10)	10 (9, 10)	0.501
Exclusive breastfeeding, % (n)**	5 (5)	0 (0)	0.024
Predominant breastfeeding, % (n)**	23 (23)	25 (25)	0.741
Mixed breastfeeding, % (n)**	66 (67)	51 (51)	0.022
Neonatal vitamin A treatment, % (n)†	53 (53)	47 (47)	0.398

Maternal characteristics	Cases (stunted) N = 101	Controls (non-stunted) N = 101	P value
Age, years; mean (SD)	24.3 (6.2)	25.0 (4.9)	0.366
Married or stable union, % (n)	97 (98)	93 (94)	0.275
Education, years; mean (SD)	9.5 (2.5)	10.2 (2.5)	0.041
Employed, % (n)	12 (12)	16 (16)	0.717
Household income per month, US dollars; median (IQR)	71 (56, 125)	82 (57, 140)	0.255
Husband's education, years; mean (SD)	10.8 (2.4)	11.3 (1.6)	0.062
Parity, median (IQR)	2 (1, 3)	2 (1, 2)	0.488
Weight, kg; mean (SD)‡	58.8 (8.8)	65.3 (12.4)	<0.001
Height, cm; mean (SD)	158.6 (6.1)	162.8 (6.8)	<0.001
Mid-upper arm circumference, cm; mean (SD)	25.7 (2.7)	26.8 (3.5)	0.020
BMI, kg/m^2^; mean (SD)‡	23.4 (3.0)	24.5 (4.9)	0.079
Maternal vitamin A treatment, % (n)†	47 (47)	54 (54)	0.325

*Defined as birth weight <2500 g.

**Detailed feeding information was collected from mothers at 6 weeks, 3 months and 6 months of age, including whether any of 22 liquids (water, juice, tea, cooking oil), milks (formula, fresh, tinned), medicines (traditional, oral rehydration solution, prescribed) or solid foods (porridge, sadza, fruit, vegetables, meat, eggs) had been given to the infant. Breastfeeding was defined as exclusive, predominant or mixed at 3 months of age, according to previously published definitions [19]. Data were not available at 3 months of age for 6 stunted and 25 non-stunted infants.

† In the ZVITAMBO trial, mother-infant pairs were randomized within 96 h of birth to one of 4 treatment groups (Aa, Ap, Pa, Pp), where ‘A’ was maternal vitamin A supplementation (400,000 IU), ‘P’ was maternal placebo, ‘a’ was infant vitamin A supplementation (50,000 IU) and ‘p’ was infant placebo. Full details of the trial have been published elsewhere [18].

‡ Maternal weight and BMI were measured 6 weeks postpartum.

WAZ: weight-for-age Z-score; HAZ: height-for-age Z-score; WHZ: weight-for-height Z-score; SD: standard deviation; IQR: interquartile range.

### Growth parameters and IGF-1 levels in cases and controls

**Figure 2:**
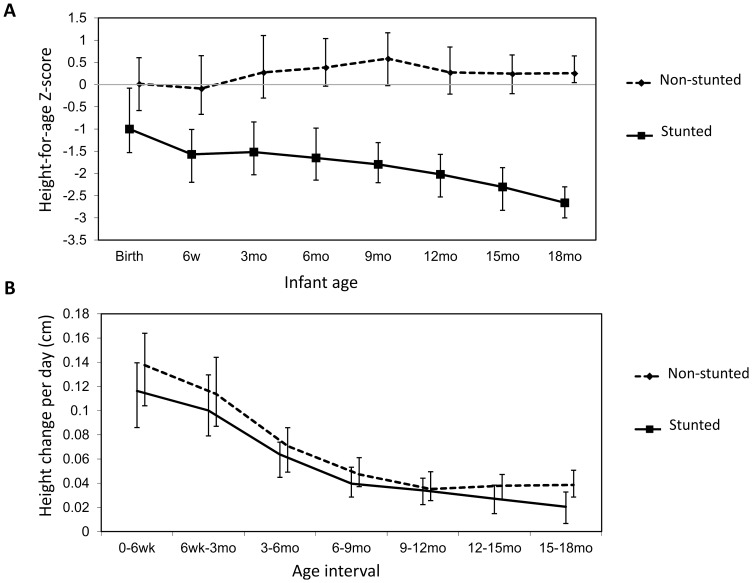
Growth in cases and controls. A: Median height-for-age Z-score (HAZ) in non-stunted (dashed line) and stunted (solid line) infants between birth and 18 months of age, with interquartile range. B: Median growth velocity in non-stunted (dashed line) and stunted (solid line) infants between birth and 18 months of age, with interquartile range. Growth velocity was calculated as height change per day, by comparing height (in centimeters) at consecutive visits and dividing by the number of days between visits.

At every time-point between birth and 18 mo, infants who were stunted at 18 mo (cases) had significantly lower height-for-age (Fig. 2A) and weight-for-age (data not shown) than infants who were not stunted at 18 mo (controls). Among cases, height-for-age Z-scores progressively declined from median (IQR) −1.00 (−1.53,−0.08) at birth to −2.02 (−2.53,−1.57) by 12 mo and −2.66 (−3.00,−2.30) by 18 mo of age. Growth velocity showed a similar pattern between groups, but was always lower in cases than controls (Fig. 2B).

**Figure 3:**
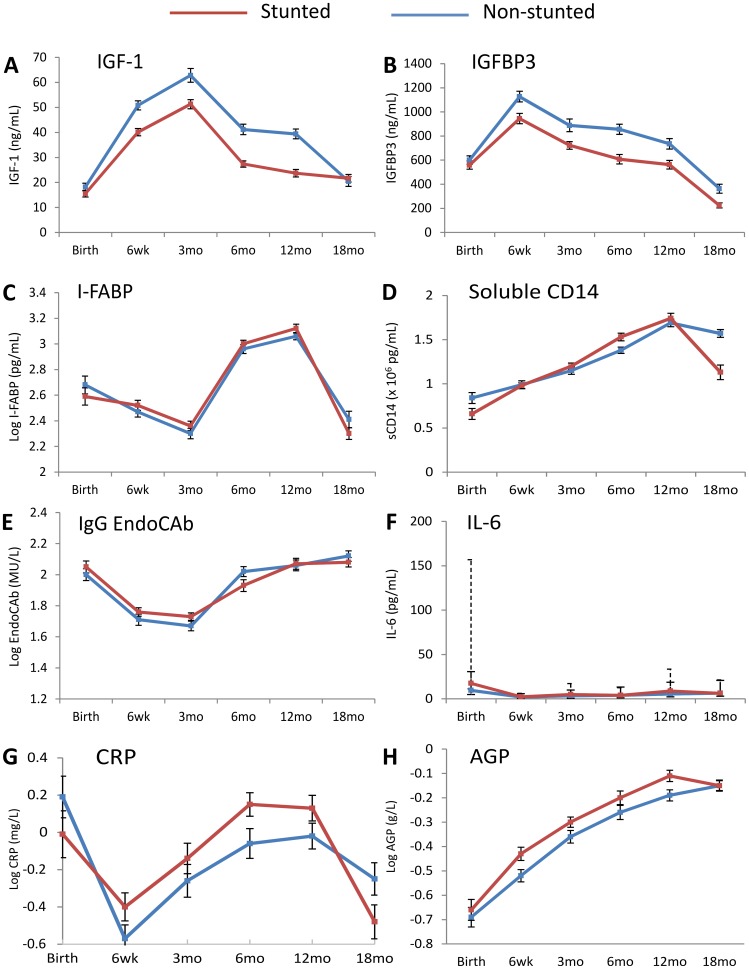
Changes in biomarkers over the first 18 months of life. Levels of **A**: Insulin-like growth factor 1 (IGF-1), **B**: IGF-binding protein 3 (IGFBP3), **C**: Intestinal fatty acid binding protein (I-FABP), **D**: Soluble CD14 (sCD14), **E**: IgG endotoxin core antibodies (EndoCAb), **F**: Interleukin-6 (IL-6), **G**: C-reactive protein (CRP) and **H**: Alpha-1 acid glycoprotein (AGP), in non-stunted (blue line) and stunted (red line) infants between birth and 18 months of age. Data shown are means with standard errors, except for IL-6, which shows medians with interquartile range (solid error bars for non-stunted infants and dashed bars for stunted infants). Mean levels, standard deviations, unadjusted and adjusted differences between cases and controls are shown in Table S1.

To test our hypothesis that stunting arises due to suppression of the growth hormone-IGF axis during infancy, we measured IGF-1 levels at each time-point between birth and 18 mo. IGF-1 levels at birth were similar in cases and controls, but from 6 weeks of age IGF-1 was significantly lower in cases than controls (adjusted mean difference (95%CI) −10.5 (−15.7, −5.3) ng/mL; Table S1). IGF-1 kinetics showed a similar pattern between groups (Fig. 3A), with an initial increase between birth and 3 mo followed by a progressive decline to 18 mo of age, when concentrations became similar in cases and controls (adjusted mean difference (95%CI) 2.0 (−2.4, 6.5) ng/mL; Table S1).

Given the clear differences in IGF-1 between cases and controls throughout infancy, we next measured levels of IGF binding protein 3 (IGFBP3), the principal binding protein of circulating IGF-1. Similar to our findings for IGF-1, levels of the binding protein were similar between groups at birth, but by 6 weeks of age IGFBP3 levels were lower in cases compared to controls (adjusted mean difference (95%CI) −211.9 (−347.3, −76.6) ng/mL) and remained lower at every time-point through 18 mo (Fig. 3B; Table S1).

### Small intestinal damage, microbial translocation and inflammation

We hypothesized that small intestinal damage, microbial translocation and inflammation would be greater in cases than controls. To evaluate gut damage, we measured plasma levels of I-FABP, a small cytoplasmic protein found in enterocytes located predominantly at the tips of small intestinal villi, which is rapidly released into the bloodstream following enterocyte damage [22]. Changes in I-FABP were very similar between groups, first declining from birth to 3 mo, then rising throughout infancy to very high levels by 12 mo (log_10_ mean (SD) 3.12 (0.32) pg/mL in cases; 3.06 (0.26) pg/mL in controls), before declining between 12–18 mo (Fig. 3C; Table S1).

To determine whether there were differences in intestinal microbial translocation between groups, we measured markers of exposure to lipopolysaccharide (LPS), a component of the gram-negative bacterial membrane. Soluble CD14, a marker of monocyte/macrophage activation produced predominantly in response to LPS stimulation, steadily rose throughout the first year of life in both groups, then fell between 12–18 mo of age (Fig. 3D). Soluble CD14 was higher in controls compared to cases at birth, but throughout infancy levels were similar between groups, until 18 mo, when levels were again higher in controls (adjusted mean difference (95%CI) −0.51 (−0.72, −0.30)×10^6^ pg/mL; Table S1). Antibodies to endotoxin core (IgG EndoCAb) were similar between groups throughout follow-up (Fig. 3E; Table S1).

To assess inflammatory status, we compared levels of IL-6, CRP and AGP over the first 18 mo of life (Fig. 3F–H; Table S1). IL-6 is produced predominantly by macrophages and monocytes and is the major pro-inflammatory cytokine that stimulates hepatic synthesis of acute phase proteins, such as CRP and AGP [23]. CRP rises and declines rapidly during an acute phase response, whereas AGP rises more slowly (more than 24 h after onset of inflammation) and remains elevated for longer [24]. IL-6 appeared strikingly elevated in cases at birth, although few infants had sufficient plasma to contribute data, so confidence intervals were wide (Fig. 3F; Table S1). IL-6 declined over the first 6 w of life then rose again throughout infancy, but levels never approached the peak seen at birth in stunted infants (Fig. 3F). From 6 weeks of age, levels of all inflammatory markers increased steadily, with cases showing higher levels of CRP and AGP than controls during the first year of life (Fig. 3G, 3H), even after adjustment for differences between groups (Table S1); however, by 18 mo of age inflammatory markers were, if anything, higher in non-stunted infants.

### Relationship between inflammation and IGF-1 levels

**Figure 4:**
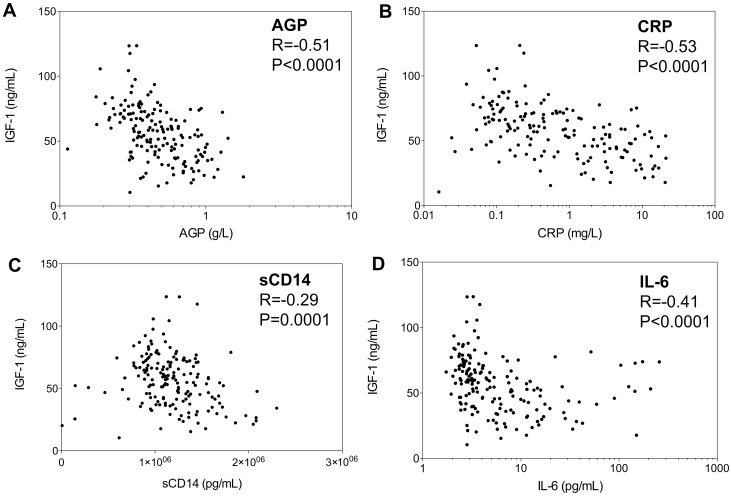
Relationships between pro-inflammatory markers and IGF-1. Associations between levels of IGF-1 and (Panel A) AGP, (Panel B) CRP, (Panel C) soluble CD14 and (Panel D) IL-6, at 3 months of age. Data for cases and controls are combined. Spearman correlations are shown.

Given the significant differences in inflammatory status between stunted and non-stunted infants we hypothesized that, even in these apparently healthy infants, low-grade inflammation inhibits hepatic production of insulin-like growth factor-1 (IGF-1), as has been shown for chronic inflammatory diseases [16], [17]. We therefore explored correlations between each inflammatory marker and IGF-1. Even at birth, infant IGF-1 levels were strongly related to levels of inflammation [AGP (R = −0.39, P<0.001), CRP (R = −0.67, P<0.001) and sCD14 (R = −0.25, P = 0.031)]. Since these associations were similar in cases and controls (data not shown), we combined groups for these analyses. Similar associations were seen at 6 w of age, and by 3 mo, the associations were even stronger (Fig. 4). When we restricted analysis to children who had a clinically normal CRP value (<5 mg/L), to exclude those with intercurrent infections driving an acute phase response at the time of sample collection, we found the same negative correlation between CRP and IGF-1 (at 6 w, R = −0.23, P = 0.011; at 3 mo, R = −0.44, P<0.001). Negative associations between each inflammatory mediator and IGF-1 were found at every subsequent time-point to 12 mo of age, but by 18 mo associations were no longer present (data not shown).

### Multivariate analysis of the associations between biomarkers and stunting

To obtain a summary measure of exposure to each biomarker over the first year of life, we calculated mean levels of IGF-1, AGP, CRP, IL-6, sCD14, EndoCAb and I-FABP between 6 w and 12 mo of age, when profiles for biomarkers were generally parallel between cases and controls. We excluded birth values because there was a high proportion of missing data (samples only available for 32% infants). We ran a multivariate model for each biomarker, adjusted for infant gender, birth weight and birth length, and maternal education, height and MUAC to calculate adjusted odds ratios for stunting at 18 mo (Table 2). Given the dynamic changes that occurred with most biomarkers between 12–18 mo of age (Fig. 3), we included the 18 mo biomarker as a covariate in the model. For each biomarker, therefore, we investigated the time-related relationships in the logistic model by including both the 6 w–12 mo mean and the 18 mo value in the model to see which, if any, contributed most to case-control differences. Higher levels of IGF-1 between 6 w–12 mo were associated with reduced odds of stunting at 18 mo (aOR 0.88 (95%CI 0.84, 0.92); P<0.001), whereas higher log_10_ levels of CRP (aOR 3.06 (95%CI 1.34, 6.99); P = 0.008) and AGP (aOR 7.87 (95%CI 0.74, 83.74); P = 0.087) between 6 w–12 mo were associated with increased odds of stunting (Table 2). There was no evidence of association between levels of I-FABP, IL-6, sCD14 or EndoCAb and stunting (Table 2).

**Table 2:** Associations between biomarkers and stunting.

Biomarker*	Unadjusted OR for stunting (95%CI) [n]	P value	Adjusted OR for stunting (95%CI) [n]**	P value
IGF-1	0.92 (0.89, 0.95) [201]	<0.001	0.88 (0.84, 0.92) [185]	<0.001
Log_10_ I-FABP	2.33 (0.80, 6.81) [194]	0.123	2.18 (0.39, 12.14) [139]	0.374
Log_10_ IgG EndoCAb	1.01 (0.27, 3.75) [202]	0.986	4.86 (0.73, 32.32) [177]	0.102
Soluble CD14 (per 10^6^)	1.52 (0.57, 4.02) [202]	0.400	3.17 (0.75, 13.47) [186]	0.118
IL-6	1.01 (1.00, 1.02) [202]	0.127	1.01 (1.00, 1.03) [185]	0.131
Log_10_ CRP	1.75 (0.95, 3.20) [202]	0.072	3.06 (1.34, 6.99) [180]	0.008
Log_10_ AGP	5.24 (0.96, 28.45) [202]	0.055	7.87 (0.74, 83.74) [173]	0.087

*A summary measure of each biomarker (mean of all values measured at 6 weeks, 3 months, 6 months and 12 months of age) was calculated for each child to reflect average exposure during the first year of life, as described in the text.

**Univariate and multivariate odds ratios of stunting at 18 months of age were calculated, using a separate model for each biomarker that adjusted for infant gender, birth weight, birth length, maternal years of schooling, maternal height and maternal mid-upper arm circumference. The 18 mo value of each biomarker was also included as a covariate in each model. Birth values of each biomarker were excluded from these models because there were insufficient data (birth samples only available for 32% infants).

IGF-1: insulin-like growth factor 1; CRP: C-reactive protein; AGP: alpha-1 acid glycoprotein; IL-6: interleukin 6; I-FABP: intestinal fatty acid binding protein; IgG EndoCAb: Immunoglobulin G antibodies to endotoxin core.

### Maternal biomarkers in cases and controls

**Figure 5:**
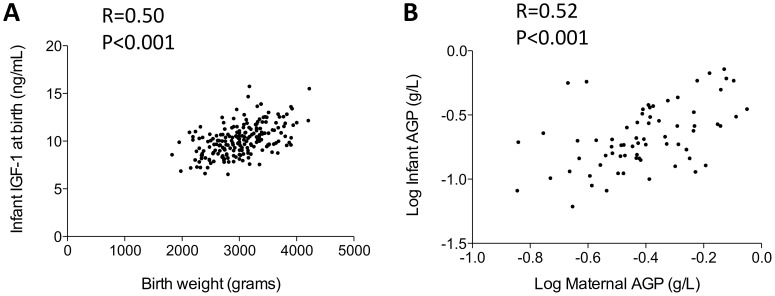
Influence of IGF-1 on birth weight and relationship between maternal and infant inflammation at birth. A: Relationship between infant levels of IGF-1, measured within 96 hours of birth, and birth weight. Spearman correlation is shown. B: Relationship between maternal and infant AGP levels measured in paired plasma samples collected within 96 hours of delivery. Spearman correlation is shown.

Because stunting began *in utero*, we next measured biomarkers in maternal plasma collected within 96 h of delivery, to test the hypothesis that maternal inflammation and IGF-1 levels influence fetal growth. Mothers of cases compared to controls had lower levels of IGF-1 (adjusted mean difference (95%CI) −21.4 (−39.8, −3.1) ng/mL; Table 3). Birth weight was associated with both maternal IGF-1 (R = 0.21, P = 0.003) and, more strongly, with infant IGF-1 (R = 0.51, P<0.0001; Fig. 5A). Birth length was not associated with maternal IGF-1 (R = 0.05, P = 0.46) but was weakly associated with infant IGF-1 levels (R = 0.23, P = 0.046). Maternal levels of IGF-1 were associated with maternal weight (R = 0.18, P = 0.029) and height (R = 0.18, P = 0.013).

**Table 3:** Biomarker levels in mothers of cases and controls.

Biomarker‡	Mothers of cases*; mean (SD) [n]†	Mothers of controls*; mean (SD) [n]†	Unadjusted difference# (95%CI)†	Adjusted difference# (95%CI)**
IGF-1 (ng/mL)	95.9 (59.6) [97]	114.3 (54.7) [100]	−18.4 (−34.5, −2.4)	−21.4 (−39.8, −3.1)
IGFBP3, ng/mL	1764.8 (904.2) [97]	1861.8 (670.6) [100]	−97.0 (−320.2, 126.2)	−118.7 (−374.9, 137.5)
Soluble CD14, ×10^6^ pg/mL	1.49 (0.63) [97]	1.49 (0.50) [100]	−0.00 (−0.16, 0.16)	−0.09 (−0.27, 0.09)
Interleukin-6, pg/mL	13.3 (9.3, 30.4) [97]	14.2 (8.6, 28.0) [100]	0.3 (−2.3, 3.1)	−0.0 (−2.7, 2.6)
CRP, log_10_ mg/L	0.97 (0.64) [45]	1.13 (0.19) [43]	−0.17 (−0.37, 0.04)	−0.04 (−0.17, 0.09)
AGP, log_10_ g/L	−0.43 (0.24) [97]	−0.40 (0.17) [100]	−0.03 (−0.09, 0.02)	−0.01 (−0.08, 0.05)

‡ Biomarkers were measured on cryopreserved plasma from blood samples collected from mothers within 96 hours of delivery.

* Mothers of cases (infants who were stunted at 18 months of age) and mothers of controls (infants who were not stunted at 18 months of age).

† Data shown are mean and standard deviation (SD) apart from IL-6, which was not normally distributed even after log transformation; data for IL-6 are therefore medians with interquartile range. For normally distributed biomarkers, unadjusted differences between means were compared using the two-sample t-test. For IL-6, unadjusted differences between medians were compared using the cendif command in Stata [21].

# Differences are biomarker values in mothers of cases minus values in mothers of controls.

** For normally distributed biomarkers, adjusted differences between means were calculated using an ordinary least-squares regression model. For IL-6, adjusted differences between medians were calculated using a median regression model. The covariates used to calculate adjusted differences in maternal biomarkers were maternal height, weight, mid-upper arm circumference and education.

IGF-1: Insulin-like growth factor-1; IGFBP3: Insulin-like growth factor binding protein 3; CRP: C-reactive protein; AGP: Alpha-1 acid glycoprotein.

We next evaluated maternal inflammatory status at birth, reasoning that mothers of cases would have higher levels of inflammation than mothers of controls. However, after adjusting for baseline differences between groups, there were no significant differences in any inflammatory biomarker between mothers of cases and controls (Table 3). Finally, we looked at the influence of maternal inflammation on infant biomarkers at birth, hypothesizing that higher levels of maternal inflammation during pregnancy may lead to higher levels of inflammation in the infant in early life. We found strong associations between maternal and infant AGP (R = 0.52, P<0.001; Fig. 5B), CRP (R = 0.46, P = 0.010) and sCD14 (R = 0.39, P<0.001), and a weak association between maternal and infant IL-6 (R = 0.31, P = 0.087) at birth.

## Discussion

We show here an association between low-grade inflammation in the first year of life and perturbation of the growth hormone-IGF axis, which is associated with stunting in apparently healthy Zimbabwean infants. We observed two important periods of poor linear growth: first, impaired intrauterine growth, which may be influenced by maternal health and *in utero* IGF-1 levels; and, second, impaired postnatal growth which was associated with chronic inflammation, starting very early in infancy. These findings highlight the importance of the first thousand days from conception to 2 years of age, during which childhood stature and ultimate developmental potential are determined, and provide an insight into the biomedical pathways that may need to be targeted to reduce the high prevalence of stunting in developing countries.

Levels of IGF-1 were generally low compared to reported values from European infant cohorts [25], [26]. However, levels of IGF-1 and its principal binding protein IGFBP3 were consistently lower among stunted compared to non-stunted infants from as early as 6 weeks of age. Whether reduced IGF-1 levels are a cause or a consequence of stunting is difficult to ascertain from our data, but given the well-characterized function of IGF-1 at growth plates [17], we speculate that lower levels are likely to mediate stunting in early life. In chronic inflammatory diseases such as juvenile idiopathic arthritis [16] and Crohn's disease [17], elevated proinflammatory cytokines mediate growth failure through a reduction in circulating IGF-1. Similarly, in our cohort of apparently healthy Zimbabwean infants, elevated inflammatory markers (even within the clinically normal range) were associated with reduced IGF-1 levels. The association between low-grade chronic inflammation and suppression of the growth hormone-IGF axis was apparent soon after delivery and may account for the decline in linear growth that occurs from birth among African and Asian infants [3]. IGF-1 levels remained lower in stunted infants throughout the first year of life, but by 18 months levels were similar between groups. This suggests that a window of opportunity may exist in infancy, during which interventions to reduce inflammation and increase IGF-1 may improve linear growth.

Although we selected cases and controls at 18 months of age, stunted infants were already growth restricted at birth. Median height-for-age Z-score at birth among these infants was −1.00 and almost one-quarter of infants in the stunted group had low birth weight. Birth weight was related to infant IGF-1 at birth, which in turn was associated with the inflammatory status of the mother-infant dyad. The infant inflammatory milieu was closely related to the level of maternal inflammation at birth. We speculate, based on these associations, that inflammation during pregnancy may ‘set’ the infant inflammatory axis, which in turn influences the level of IGF-1 in early life. Optimizing the health of pregnant women may be essential to impact antenatal and postnatal stunting. Infants who became stunted were born to mothers who themselves were shorter than mothers of non-stunted infants. However, it was striking that over one-quarter of mothers of cases were overweight or obese. The relationship between maternal and fetal nutritional status is therefore complex and requires further investigation, particularly in view of the emerging obesity epidemic in countries that are experiencing the nutrition transition [27], [28].

Intestinal damage during infancy was common in both cases and controls. There is no single, well-validated enteropathy biomarker available; most prior studies have used non-invasive markers of intestinal permeability because intestinal biopsies are difficult to obtain in childhood [15], [29]. We used plasma I-FABP as a marker of small intestinal damage; this is the first study to our knowledge that has used I-FABP to evaluate environmental enteropathy in any setting. I-FABP is a reliable marker of enteropathy in children with celiac disease [30], which is similarly characterized by villous atrophy, intestinal inflammation and increased intestinal permeability; levels correlate with biopsy disease stage and decline rapidly on introduction of a gluten-free diet [30]. In our study, I-FABP rose between 3–12 months of age to exceptionally high levels (mean 1236 pg/mL at 12 mo) compared to values reported for healthy European/US children or adults using the same assay (mean 20–183 pg/mL) [30], [31], [32], [33]; values even exceeded those in European children with celiac disease (mean 458–785 pg/mL) [30], [33], suggesting that extensive small intestinal damage is the norm among children living in impoverished conditions. The amount of intestinal damage was virtually indistinguishable between cases and controls throughout infancy and levels of I-FABP were not associated with stunting in multivariate analysis. The timing of gut damage is consistent with previous findings from our group [19] that non-breast milk liquids and complementary foods are frequently introduced into the diet very early in infancy. Non-exclusive breastfeeding may lead to feco-oral transmission of bacteria among infants living in conditions of poor sanitation and hygiene; frequent exposure to potentially pathogenic organisms likely drives the T-cell mediated enteric inflammation that characterizes EE [14]. Interestingly, there was a striking reduction in I-FABP in both groups between 12–18 months, suggesting mucosal repair may occur beyond infancy, for reasons that are unclear. Taken together, we show here that high levels of small intestinal damage occur during infancy, but we did not find evidence of association with stunting. Because appropriate samples were not available, we were unable to investigate whether a more extensive panel of markers, including measures of intestinal inflammation, permeability and repair, would establish a link between enteropathy and stunting, as has been demonstrated in recent studies from other cohorts [34], [35], [36], [37].

There are several limitations to this study. First, women were recruited shortly after delivery, so we were unable to characterize maternal inflammation or IGF-1 levels during pregnancy, and biomarker levels may have been influenced by parturition; however, we assume that levels of biomarkers measured shortly after delivery at least partly reflect the antenatal milieu. Second, we did not measure LPS levels because plasma was not collected into endotoxin-free tubes. Rising levels of soluble CD14 and EndoCAb during infancy indirectly indicate chronic exposure to LPS [38], but the source of endotoxin was not studied. Systemic endotoxin exposure can occur via the gut or the lung, and airborne endotoxin exposure can be high where animal dung is used as cooking fuel [39]. However, use of dung as fuel is rare in Zimbabwe and among Gambian infants there was a high correlation between intestinal permeability and plasma endotoxin [29]. These observations suggest that the most likely that the source of endotoxin in these urban Zimbabwean infants was microbial contamination of the environment, and a permeable gut (i.e. EE) was the major pathway of exposure. Third, this is an observational study and it is difficult to make inferences about causality; nonetheless, our findings are consistent with the hypothesis that enteropathy occurs early in life and that chronic inflammation is a mediator of poor growth in infancy [10]. Finally, the generalizability of our findings is uncertain. Due to the case-control design of this study, and the need to restrict enrolment to infants with available anthropometry and stored samples, study subjects may differ in several respects to the wider group of infants in developing countries. We chose non-stunted infants based on a Z-score cut-off of >−0.5 to maximize the distinction between cases and controls, without over-selecting for potential outliers with good growth; however, selection of controls is potentially subject to bias. Prospective studies of other cohorts are needed to further investigate the mechanisms of stunting suggested in this case-control study.

In summary, our data suggest that stunting is influenced by both maternal and infant factors. Antenatally, maternal nutritional and inflammatory status may impact fetal growth, leading to intrauterine stunting and low birth weight; postnatally, low-grade inflammation early in life is associated with stunting. The reasons for higher levels of CRP and AGP in stunted compared to non-stunted infants are unclear and require further investigation. We show here that infants have extensive small intestinal injury and evidence of exposure to LPS during infancy, but we found no evidence that gut damage was associated with stunting. However, future studies of prospective cohorts should use a more extensive panel of markers to characterize enteric dysfunction, as recently proposed [40]. Whether small intestinal damage can be prevented during infancy is currently being evaluated in several cluster-randomized trials of improved sanitation and hygiene, including WASH Benefits in Kenya (NCT01704105) and Bangladesh (NCT01590095) and the Sanitation Hygiene Infant Nutrition Efficacy (SHINE) trial in Zimbabwe (NCT01824940). These trials are testing the hypothesis that reducing feco-oral transmission of bacteria during infancy will prevent environmental enteropathy and reduce the prevalence of stunting in developing countries. Our study suggests that interventions to ameliorate the inflammatory milieu need to be targeted very early during the first thousand days to promote healthy growth.

## Supporting Information

Table S1 **Biomarker levels in stunted and non-stunted infants.** Biomarker levels with unadjusted and adjusted differences in values between stunted and non-stunted infants, between birth and 18 months of age. Number of samples available for each measurement are shown in square brackets.
(DOCX)
