# Supplementary material for: Stunting Is Characterized by Chronic Inflammation in Zimbabwean Infants
Source: PLoS One. 2014 Feb 18;9(2):e86928. doi: 10.1371/journal.pone.0086928 (PMC3928146; doi:10.1371/journal.pone.0086928)
Supplement: Table S1 — Biomarker levels in stunted and non-stunted infants. Biomarker levels with unadjusted and adjusted differences in values between stunted and non-stunted infants, between birth and 18 months of age. Number of samples available for each measurement are shown in square brackets. (DOCX) [file pone.0086928.s001.docx]

**Table S1: Biomarker levels in stunted and non-stunted infants**

| **Biomarker** | **Cases (stunted)*; mean (SD) [n] †** | **Controls (non-stunted)*; mean (SD) [n] †** | **Unadjusted difference # (95%CI) †** | **Adjusted difference (95%CI)**** |
| --- | --- | --- | --- | --- |
| **IGF-1 (ng/mL)** |  |  |  |  |
| Birth | 15.5 (7.6) [34] | 18.2 (9.5) [39] | -2.7 (-6.7, 1.4) | 0.7 (-4.1, 5.4) |
| 6 weeks | 40.1 (12.2) [71] | 50.8 (13.8) [57] | -10.7 (-15.2, -6.1) | -10.5 (-15.7, -5.3) |
| 3 months | 51.3 (18.2) [99] | 62.8 (23.3) [72] | -11.5 (-17.8, -5.3) | -12.6 (-19.8, -5.5) |
| 6 months | 27.4 (11.8) [91] | 41.2 (18.3) [78] | -13.8 (-18.4, -9.2) | -13.5 (-18.7, -8.3) |
| 12 months | 23.7 (12.2) [65] | 39.4 (16.3) [68] | -15.8 (-20.7, -10.8) | -16.0 (-21.7, -10.2) |
| 18 months | 21.7 (14.6) [101] | 20.4 (14.1) [99] | 1.3 (-2.7, 5.3) | 2.0 (-2.4, 6.5) |
|  |  |  |  |  |
| **IGFBP3 (ng/mL)** |  |  |  |  |
| Birth | 559.3 (196.5) [32] | 600.8 (217.1) [39] | -41.5 (-140.5, 57.5) | -34.5 (-161.8, 92.9) |
| 6 weeks | 944.4 (356.9) [70] | 1126.9 (336.2) [57] | -182.5 (-305.3, -59.7) | -211.9 (-347.3, -76.6) |
| 3 months | 722.0 (316.9) [97] | 888.7 (432.0) [68] | -166.7 (-281.8, -51.6) | -146.5 (-280.5, -12.6) |
| 6 months | 607.4 (326.6) [69] | 856.0 (324.4) [59] | -248.6 (-362.9, -134.4) | -232.9 (-361.4, -104.4) |
| 12 months | 562.3 (340.7) [92] | 735.3 (405.5) [87] | -173.0 (-283.2, -62.7) | -154.3 (-281.1, -27.6) |
| 18 months | 224.3 (204.4) [95] | 363.1 (353.7) [91] | -138.8 (-221.9, -55.7) | -133.4 (-230.2, -36.6) |
|  |  |  |  |  |
| **I-FABP (log_10_ pg/mL)** |  |  |  |  |
| Birth | 2.59 (0.33) [24] | 2.68 (0.42) [37] | -0.09 (-0.30, 0.11) | -0.14 (-0.40, 0.12) |
| 6 weeks | 2.52 (0.32) [63] | 2.47 (0.29) [49] | 0.05 (-0.65, 0.17) | 0.11 (-0.19, 0.24) |
| 3 months | 2.36 (0.32) [77] | 2.30 (0.29) [54] | 0.06 (-0.05, 0.17) | 0.03 (-0.10, 0.16) |
| 6 months | 3.00 (0.24) [73] | 2.96 (0.24) [47] | 0.04 (-0.05, 0.13) | 0.05 (-0.05, 0.15) |
| 12 months | 3.12 (0.32) [88] | 3.06 (0.26) [83] | 0.07 (-0.02, 0.15) | 0.09 (-0.02, 0.19) |
| 18 months | 2.30 (0.41) [84] | 2.41 (0.55) [74] | -0.11 (-0.26, 0.04) | -0.14 (-0.31, 0.03) |
|  |  |  |  |  |
| **Soluble CD14 (x10^6^ pg/mL)** |  |  |  |  |
| Birth | 0.66 (0.36) [34] | 0.84 (0.38) [39] | -0.17 (-0.35, 0.00) | -0.25 (-0.48, -0.03) |
| 6 weeks | 0.98 (0.30) [71] | 0.99 (0.34) [57] | -0.01 (-0.12, 0.10) | 0.03 (-0.10, 0.15) |
| 3 months | 1.20 (0.35) [100] | 1.15 (0.35) [73] | 0.05 (-0.05, 0.16) | 0.08 (-0.04, 0.20) |
| 6 months | 1.53 (0.44) [100] | 1.38 (0.33) [86] | 0.14 (0.03, 0.26) | 0.17 (0.05, 0.30) |
| 12 months | 1.74 (0.60) [101] | 1.69 (0.42) [98] | 0.05 (-0.09, 0.20) | 0.10 (-0.07, 0.27) |
| 18 months | 1.13 (0.83) [101] | 1.57 (0.43) [99] | -0.43 (-0.62, -0.25) | -0.51 (-0.72, -0.30) |
|  |  |  |  |  |
| **EndoCAb (log­_10_ MU/mL)** ‡ |  |  |  |  |
| Birth | 2.05 (0.20) [27] | 2.00 (0.22) [34] | 0.05 (-0.06, 0.16) | 0.08 (-0.05, 0.22) |
| 6 weeks | 1.76 (0.22) [67] | 1.71 (0.04) [57] | 0.04 (-0.05, 0.13) | 0.10 (-0.00, 0.20) |
| 3 months | 1.73 (0.23) [88] | 1.67 (0.25) [65] | 0.06 (-0.02, 0.14) | 0.11 (0.02, 0.19) |
| 6 months | 1.93 (0.33) [75] | 2.02 (0.25) [62] | -0.09 (-0.19, 0.01) | -0.05 (-0.16, 0.06) |
| 12 months | 2.07 (0.34) [94] | 2.06 (0.30) [83] | 0.02 (-0.08, 0.11) | 0.04 (-0.07, 0.14) |
| 18 months | 2.08 (0.30) [95] | 2.12 (0.32) [93] | -0.04 (-0.13, 0.05) | 0.00 (-0.10, 0.10) |
|  |  |  |  |  |
| **CRP (log_10_ mg/L)** |  |  |  |  |
| Birth | -0.01 (0.70) [31] | 0.19 (0.69) [37] | -0.10 (-0.27, 0.07) | -0.35 (-0.76, 0.07) |
| 6 weeks | -0.40 (0.63) [70] | -0.57 (0.55) [56] | 0.18 (-0.03, 0.39) | 0.19 (-0.04, 0.42) |
| 3 months | -0.14 (0.80) [95] | -0.26 (0.74) [71] | 0.12 (-0.12, 0.36) | 0.19 (-0.08, 0.47) |
| 6 months | 0.15 (0.61) [95] | -0.06 (0.73) [83] | 0.21 (0.01, 0.41) | 0.27 (0.05, 0.50) |
| 12 months | 0.13 (0.66) [91] | -0.02 (0.67) [94] | 0.15 (-0.04, 0.35) | 0.28 (0.06, 0.50) |
| 18 months | -0.48 (0.89) [96] | -0.25 (0.86) [98] | -0.22 (-0.47, 0.02) | -0.24 (-0.53, 0.04) |
|  |  |  |  |  |
| **AGP (log_10_ g/L)** |  |  |  |  |
| Birth | -0.66 (0.25) [33] | -0.69 (0.25) [37] | 0.03 (-0.09, 0.15) | -0.04 (-0.19, 0.11) |
| 6 weeks | -0.43 (0.23) [71] | -0.52 (0.19) [57] | 0.09 (0.02, 0.16) | 0.08 (-0.00, 0.16) |
| 3 months | -0.30 (0.20) [94] | -0.36 (0.22) [69] | 0.06 (-0.10, 0.12) | 0.04 (-0.04, 0.12) |
| 6 months | -0.20 (0.23) [70] | -0.26 (0.22) [59] | 0.05 (-0.03, 0.13) | 0.03 (-0.06, 0.12) |
| 12 months | -0.11 (0.22) [94] | -0.19 (0.21) [85] | 0.07 (0.01, 0.14) | 0.09 (0.02, 0.17) |
| 18 months | -0.15 (0.19) [94] | -0.15 (0.22) [92] | 0.01 (-0.05, 0.07) | -0.01 (-0.08, 0.06) |
|  |  |  |  |  |
| **Interleukin-6 (pg/mL)** |  |  |  |  |
| Birth | 17.5 (6.2, 139.3) [15] | 9.6 (4.7, 21.2) [16] | 5.4 (-3.4, 124.8) | 14.2 (-22.0, 50.3) |
| 6 weeks | 2.4 (1.7, 3.8) [44] | 2.2 (1.8, 3.6) [41] | 0.1 (-0.4, 0.6) | 0.3 (-0.5, 1.1) |
| 3 months | 4.9 (2.8, 12.4) [101] | 3.4 (2.8, 6.6) [72] | 0.6 (-0.0, 2.1) | 0.9 (-0.8, 2.7) |
| 6 months | 4.0 (2.8, 8.6) [95] | 4.0 (2.8, 8.4) [79] | 0.1 (-0.7, 0.8) | -0.1 (-1.2, 1.0) |
| 12 months | 8.9 (4.1, 24.6) [100] | 5.4 (3.1, 13.4) [93] | 1.6 (0.2, 3.5) | 2.8 (-0.1, 5.7) |
| 18 months | 6.2 (3.2, 15.2) [101] | 6.4 (3.4, 14.4) [98] | 0.0 (-1.0, 1.3) | -1.2 (-4.0, 1.6) |

**#** Differences are biomarker values in cases (stunted) minus controls (non-stunted).

*Cases and controls were selected based on height at 18 months (cases: height-for-age Z-score <-2.0; controls: height-for-age Z-score >-0.5), and cryopreserved plasma samples from birth through 18 months were then retrieved and tested for biomarkers. Samples collected within 96 hours of birth were available for a subset (32%) of infants.

† Data shown are mean and standard deviation (SD) apart from IL-6, which was not normally distributed even after log transformation; data for IL-6 are therefore medians with interquartile range. For normally distributed biomarkers, unadjusted differences between means were compared using the two-sample t-test. For IL-6, unadjusted differences between medians were compared using the cendif command in Stata [21].

** For normally distributed biomarkers, adjusted differences between means were calculated using an ordinary least-squares regression model. For IL-6, adjusted differences between medians were calculated using a median regression model. The covariates used to calculate adjusted differences in infant biomarkers were gender, birth weight, maternal mid-upper arm circumference and maternal education.

‡ IgG Endotoxin core antibody (EndoCAb) is measured in arbitrary standard median units (MU), based on medians of ranges for 1000 healthy adults, as per manufacturer’s instructions (Hycult Inc).

IGF-1: Insulin-like growth factor-1; IGFBP3: Insulin-like growth factor binding protein 3; I-FABP: Intestinal fatty acid binding protein; EndoCAb: IgG Endotoxin core antibody; CRP: C-reactive protein; AGP: Alpha-1 acid glycoprotein.
